# Response to Letter to the Editor From Luis Del Carpio-Orantes: [Impact of Surreptitious Glucocorticoids in Over-the-counter Arthritis Supplements]

**DOI:** 10.1210/jendso/bvaf045

**Published:** 2025-03-14

**Authors:** Kevin S Wei, Carolina R Hurtado, Trevor E Angell

**Affiliations:** Department of Medicine, Los Angeles General Medical Center, Los Angeles, CA 90033, USA; Division of Endocrinology, Los Angeles General Medical Center, Los Angeles, CA 90033, USA; Division of Endocrinology and Diabetes, Keck Medicine of USC, Los Angeles, CA 90033, USA

**Keywords:** Cushing's syndrome, Artri King, adrenal insufficiency, cortisol, glucocorticoid-induced, supplements

To the Editor: We recognize the insightful comments in the letter of Dr. Del Carpio-Orantes [1] and appreciate his dedicated efforts to protect people from the deleterious health effects of unregulated supplements. He has been a particular leader sounding the alarm on Artriking, as well as similar glucocorticoid containing products, that can cause Cushing's syndrome and adrenal insufficiency. Dr. Del Carpio-Orantes notes that these supplements frequently do not indicate that any glucocorticoid is present. We suggest that even if glucocorticoids were listed, users are unlikely to recognize them as a potentially harmful constituent, leading to the insidious onset of adverse effects even while there may be some anti-inflammatory relief. Indeed, we have observed clinically that when patients experience weakness, nausea, and/or lightheadedness (all indicative of adrenal insufficiency) upon discontinuation of the supplement, it only further erroneously reinforces the supposed benefit of the product, thereby perpetuating its use.

Dr. Del Carpio-Orantes calls attention to the origin of many of these products in Mexico and their spread to the United States and beyond. Interestingly, the existing published reports on these supplements provide support for this statement. Assessing the studies included in our literature review along with our own case series [2], 7 of 8 reports come from states sharing a border with Mexico: Texas, Arizona, and California. Furthermore, we acknowledge another series of similar patients in southern California that has been recently reported [3].

We concur with Dr. Del Carpio-Orantes that these cases may be just be the tip of the iceberg. Past data indicate that over one-third of US adults use some alternative health method and spend over $10 billion on supplements [4]. Those data are now more than 10 years old, and the current environment of misinformation in nontraditional media, as well as internet consumerism, has likely resulted in these products spreading farther and more easily than ever before.

We conclude by reiterating Dr. Del Carpio-Orantes’ direction that clinicians should remain alert to the possibility of supplements causing surreptitious glucocorticoid exposure and educate patients about their existence and potential effects.

## Disclosures

The authors have nothing to disclose.
