# Correction to: “Response to Letter to the Editor From Luis Del Carpio-Orantes: [Impact of Surreptitious Glucocorticoids in Over-the-counter Arthritis Supplements]”

**DOI:** 10.1210/jendso/bvaf092

**Published:** 2025-06-03

In the above-named article by Wei KS, Hurtado CR, and Angell TE (*J. Endocr Soc.* 2025; 9(5); doi: 10.1210/jendso/bvaf045), there was an error in reference 1.

In the Letter Response as originally published, reference 1 appeared as “Del Carpio-Orantes L. Letter to editor: impact of surreptitious glucocorticoids in over-the-counter arthritis supplements. *J Endocr Soc*. 2024;9(2):bvae227. The corrected reference 1 is “Del Carpio-Orantes L. Letter to editor: impact of surreptitious glucocorticoids in over-the-counter arthritis supplements. *J Endocr Soc*. 2025; 9(6): bvaf044, https://doi.org/10.1210/jendso/bvaf044

The Letter Response has been corrected online.

doi: 10.1210/jendso/bvaf045

